# Critically ill patients with acute cholecystitis are at increased risk for extensive gallbladder inflammation

**DOI:** 10.1186/s13017-015-0054-1

**Published:** 2015-12-01

**Authors:** Marios Papadakis, Peter C. Ambe, Hubert Zirngibl

**Affiliations:** Helios Klinikum Wuppertal, Department of Surgery II, Witten – Herdecke University, Heusner Str. 40, 42283 Wuppertal, Germany

**Keywords:** Acute cholecystitis, Critically ill patients, Laparoscopic cholecystectomy

## Abstract

**Background:**

Acute cholecystitis is a common diagnosis and surgery is the standard of care for young and fit patients. However, due to high risk of postoperative morbidity and mortality, surgical management of critically ill patients remains a controversy. It is not clear, whether the increased risk of perioperative complications associated with the management of critically ill patients with acute cholecystitis is secondary to reduced physiologic reserve per se or to the severity of gallbladder inflammation.

**Methods:**

A retrospective analysis of prospectively collected data of patients undergoing laparoscopic cholecystectomy for acute cholecystitis in a university hospital over a three-year-period was performed. The ASA scores at the time of presentation were used to categorize patients into two groups. The study group consisted of critically ill patients with ASA 3 and 4, while the control group was made up of fit patients with ASA 1 and 2. Both groups were compared with regard to perioperative data, postoperative outcome and extent of gallbladder inflammation on histopathology.

**Results:**

Two hundred and seventeen cases of acute cholecystitis with complete charts were available for analysis. The study group included 67 critically ill patients with ASA 3 and 4, while the control group included 150 fit patients with ASA 1 and 2. Both groups were comparable with regard to perioperative data. Histopathology confirmed severe cholecystitis in a significant number of cases in the study group compared to the control group (37 % vs. 18 %, *p* = 0.03). Significantly higher rates of morbidity and mortality were recorded in the study group (p < 0.05). Equally, significantly more patients from the study group were managed in the ICU (40 % vs. 8 %, *p* = 0.001).

**Conclusion:**

Critically ill patients presenting with acute cholecystitis are at increased risk for extensive gallbladder inflammation. The increased risk of morbidity and mortality seen in such patients might partly be secondary to severe acute cholecystitis.

## Background

Acute cholecystitis (AC) is usually an indication for surgery. Current evidence suggests early cholecystectomy to be superior to interval or delayed cholecystectomy with regard to outcome and cost of treatment [1–8]. However, the heterogeneity of patients with acute cholecystitis (AC) makes it almost impossible to standardize treatment. Thus, while healthier and “fit for surgery” candidates are generally surgically managed, controversy exists in the management of elderly and critically ill patients [9–17]. Medical management with antibiotics, fasting, antiemetic and pain medication as well as percutaneous gallbladder aspiration or drainage have been employed as alternative treatment options [12, 16, 18–21]. These options have either been used as a definite means of treatment or as a bridge to surgery after cooling down the acute inflammation.

Patients with reduced physiologic reserves, that is critically ill and elderly patients, have been shown to be at increased risk for postoperative morbidity following gallbladder surgery for acute cholecystitis. However, it is not clear whether or not this increased risk of postoperative morbidity is secondary to reduced physiologic reserve per se or to the extent of gallbladder inflammation. The aim of this study therefore was to investigate whether or not critically ill patients with acute cholecystitis are at increased risk for extensive gallbladder inflammation.

## Patients and methods

Following the approval of the ethic committee of the Witten – Herdecke University, a retrospective analysis of a prospectively maintained database of patients undergoing surgery for acute cholecystitis was performed. A written consent was obtained from each patient included. The data of all consecutive patients with AC scheduled for surgery from January 2012 until December 2014 were extracted by two independent investigators.

Patients were admitted following presentation in the emergency department or following referral from the department of internal medicine. Acute cholecystitis was suspected in patients presenting with pain to the right upper quadrant. The diagnosis was confirmed following findings from abdominal ultrasound sonography and blood chemistry as outlined in the Tokyo Guidelines [22, 23].

As part of our institutional standards, all patients with AC were put on i.v. antibiotics usually Tazobactam. Surgery was scheduled as soon as possible depending on the presence or absence of comorbidities needing special consultation and correction. The time interval between admission and surgery was calculated for each patient.

Laparoscopic cholecystectomy (LC) is a standard procedure in our department. In all cases LC was performed using four trocars. Surgery began with the placement of an 11 mm trocar just above the umbilicus after a mini-laparotomy. Occasionally, a Veress needle was used to create pneumoperitoneum. The maximum intraabdominal pressure was set at 12 mmHg. Surgery proceeded with the placement of a 12 mm trocar in the epigastrium slightly to the left of middle line and two 5 mm trocars in the right upper abdomen under visual control. The leading surgeon was either an attending surgeon with expertise in laparoscopic surgery, a fellow or a senior resident under supervision. Single shot antibiotic was administered before incision depending on the time interval between the last antibiotic application and begin of surgery. The gallbladder was removed via the supraumbilical mini-laparotomy using an endobag. Histopathology was performed in all cases.

The data of each patient was put into the database after discharge from the hospital. Demographic data including sex, age, body mass index (BMI) and medical comorbidity score as defined by the American Society of Anesthesiologists (ASA) at the time of surgery were retrieved for each patient. Preoperative data including; white blood count (WBC), c - reactive protein (CRP), platelet count and findings from abdominal ultrasound sonography were recorded for each patient. Perioperative data including surgical procedure, the course of surgery and the duration of surgery (time from incision to suture) were registered in each case. Postoperative data including; complications, management in the intensive care unit and the length of stay (LOS) were recorded for each case. The final histopathology records were consulted for the extent of gallbladder inflammation. The following terminologies were used to define the extent of gallbladder inflammation:

- edematous choleystitis was diagnosed in cases with gallbladder wall edema with interstitial fluid, dilated capillaries and lymphatics.
- necrotizing cholecystitis was diagnosed following the presence of scattered superficial necrosis of the gallbladder wall
- gangrenous cholecystitis was characterized by loss of mucosal lining and vascular architecture with profuse inflammation changes secondary influx of inflammatory cells [24].

The terminologies used to describe the extent of gallbladder inflammation on histopathology in this series are primarily german and correspond grossly with English terminologies used elsewhere [25]. Edematous cholecystitis was considered „uncomplicated while empyematous, necrotizing and gangrenous cholecystitis were referred to as „complicated cholecystitis“.

In this study, a critically ill patient was defined as any patient with reduced physiologic reserve presenting with acute cholecystitis. Although the ASA score relies mostly on chronic conditions, it does account to some degree for the acute illness. Thus critically ill patients were defined as those with relevant chronic conditions presenting with AC [26]. The study group therefore consisted of patients with ASA 3 and 4, while the control group was made up of patients with ASA 1 and 2.

The Statistical Package for Social Science (SPSS®), IBM, version 22 was employed to analyze the collected data. Continuous variables were described using absolute case numbers and percentages. Since the data were not normally distributed, central tendencies were described using median values with the corresponding interquartile ranges with a 95 % confidence interval. Statistical significances were calculated using chi square test with the level of significance placed at *p* < 0.05.

Both groups would be compared with regard to perioperative findings and postoperative outcomes. Primary outcome was the extent of gallbladder inflammation on histopathology. Secondary outcomes included the duration of surgery, the rate of conversion, postoperative morbidity as defined by Clevian and Dindo [27], the need of ICU management and the LOS.

## Results

**Fig. 1:**
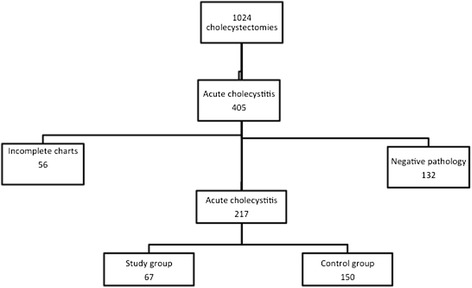
Distribution of the study population. The study group consisted of 67 critically ill patients while the control group was made up of 150 healthier patients

Within the period of investigation, 1024 cholecystectomies were performed in our department. The indication for surgery was AC in 405 cases. After excluding cases with negative pathology (cases with chronic cholecystitis presenting with acute biliary colics) and incomplete data (ASA, BMI and gallbladder wall thickness per ultrasound sonography), 217 cases of AC were included for analysis, Fig. 1. The demographic features of the cases included are summarized in Table 1. The study group was significantly older compared to the control group, *p* = 0.02.

**Table 1:** Demographic characteristics

Characteristics	Study group	Control group	*p*-value
Number of cases	67	150	/
Sex: female	28 (42.4 %)	66 (44.0 %)	0.44
male	38 (57.6 %)	84 (56.0 %)	
Median Age (yrs)	76.0	59.0	0.02
Interquartile range	19.0	25.0	
Median BMI (kg/m^2^)	27.1	27.4	0.88
Interquartile range	6.5	5.7	

The study group was significantly older compared to the control group. *Yrs* years, *BMI* body mass index

Cardiovascular disorders including high blood pressure, ischemic heart disease, hypertensive heart disease, atrial fibrillation and peripheral artery disease constituted the leading concomitant disorders in the study and were recorded in 55 cases (82 %). Type 2 diabetes was present in 27 cases (40 %). Chronic obstructive pulmonary disease was present in 18 cases (26.8 %) while chronic kidney disease was seen in 16 cases (23 %).

The perioperative characteristics in this study are presented in Table 2. There was no significant difference amongst both groups with regard to perioperative findings. Early cholecystectomy was performed within 72 h of symptom onset in over 80 % (54/67) of the study group and in about 91 % (137/150) of the control group. Delayed cholecystectomy, that is surgery after 72 h of symptom onset was performed in 19.4 % (13 cases) of the study group and in 8.7 % (13 cases) of the control group. This difference was statistically significant, *p* = 0.02.

**Table 2:** Perioperative data

Features	Study group	Control group	*p*-value
Median gallbladder wall thickness	6.0	5.0	0.26
Interquartile range	11.0	4.0	
WBC/ul	12.6	10.8	0.50
Interquartile range	8.5	6.8	
CRP	10.5	3.7	0.14
Interquartile range (mg/dl)	20.5	12.9	
Median time to surgery	24.0	24.0	0.10
Interquartile range (h)	60.0	42.0	
Median duration of surgery	98.0	87.0	0.28
Interquartile range (min)	63.0	58.0	
Conversion to open surgery	3/66 (4.5 %)	11/150 (7.3 %)	0.43

Both groups were comparable in terms of perioperative data

*h* hours, *min* minutes, *CRP* c – reactive protein, *WBC* white blood count

Conversion from laparoscopic to open surgery was necessary in 14 cases (6.5 %). While conversion was necessary in just three cases (4.5 %) in the study group, 11 cases (7.3 %) were converted in the control group. This difference was not statistically significant, *p* = 0.42. All converted cases were monitored in the ICU.

Complications were recorded in 25 (11.5 %) cases in the entire study population. These included 14 (20.9 %) cases in the study group and 11 (7.3 %) cases in the control group. This difference was statistically significant, *p* = 0.004). The postoperative outcomes of both groups are summarized in Table 3, while the complications recorded in both groups are presented in Table 4.

**Table 3:** Postoperative data

Features	Study group	Control group	P - value
ICU admission	27/67 (40.3 %)	12/150 (8.0 %)	0.001
Morbidity rate	14/67 (20.9 %)	11/150 (7.3 %)	0.004
Median length of stay Interquartile range (d)	6.0	4.0	0.001
	6.0	3.0	

The need for ICU management, the rate of morbidity and the median length of postoperative hospital stay were significantly higher in the study group. d: days

**Table 4:** Complications

Complications	Study group	Control group
Grade I	4	4
Grade II	2	0
Grade III	5	6
Grade IV	1	0
Grade V	2	1

Postoperative complications according to the classification by Dindo et al. [27]

Study group:

I: delayed bowel movement 2x, superfacial wound bleeding 1x, wound dehiscence 1x

II: Pneumonia 2x

III: Bile leak 2 x, bilioma drainage 1x, bleeding 1x, subphrenic hematoma 1x

IV: Acute renal failure (Dialysis)

V: Mortality 1x (pulmonary embolism), 1 x pulmonary failure

Control group:

I: wound dehiscence 2x, wound infection 1x, small bilioma 1x

III: Subphrenic abscess 3x, Hematoma 1x, bile duct injury 2x

V: Mortality 1x (myocardial infarction)

ICU management was necessary in 40.9 % of cases in the study group, while only 8 % of cases in the control group required ICU admission. This difference was statistically significant, *p* = 0.001.

Uncomplicated cholecystitis was diagnosed in 63.6 % (42/67) of study group and in 82.0 % (123/150) of the control group following histopathology. Significantly (*p* = 0.03) more complicated gallbladder inflammation was evident in the study group (25/76 : 37.3 %) compared to the control group (27/150 : 18 %), Table 5. Three cases of mortality (1.4 %) were recorded in this series including two cases (1.3 %) in the study group and one case (1.5 %) in the control group. This difference was not statistically significant, *p* = 0.172.

**Table 5:** Histopathology

Histopathology	Study group	Control group	P -value
Uncomplicated acute cholecystitis	42/67 (62.6 %)	123/150 (82.0 %)	0.42
Empyematous cholecystitis	5/67 (7.5 %)	4/140 (2.7 %)	
Gangrenous cholecystitis	14/67 (20.9%)	14/150 (9.3 %)	0.02
Necrotizing cholecystitis	5/67 (7.5 %)	9/150 (6.0 %)	

Uncomplicated cholecystitis was seen in a vast majority of cases in both groups. Complicated cholecystitis was diagnosed in significantly more patients from the study group, *p* = 0.02

## Discussion

This study was designed to investigate whether or not critically ill patients with acute cholecystitis could be at risk for extensive gallbladder inflammation. The charts of 217 patients undergoing laparoscopic cholecystitis for acute cholecystitis within a three-year-period in a university hospital were retrospectively reviewed. Patients with relevant chronic conditions (ASA scores > 2) presenting with AC were classified as critically ill. The study group consisted of 67 critically ill patients with ASA > 2, while the control group was made up of 150 fit patients with ASA ≤ 2. Although the study group was significantly older than the control group, both groups were comparable with regard to perioperative data. The rates of morbidity and mortality were significantly higher in the study group. Equally, significantly more patients from the study group required ICU management following surgery. Histopathology confirmed extensive gallbladder inflammation in the form of gangrenous, necrotizing and empyematous cholecystitis in a significant number of cases from the study group.

Although acute cholecystitis is a common illness, the great divergence in the clinical course and possible concomitant medical conditions make it extremely difficult to standardize management. While early surgery is generally accepted as the standard of care for young and fit patients, the management of critically ill patients has been vague and is still a cause of controversy due to high rates of morbidity and mortality associated with the surgical management of such patients [4, 5, 9, 12, 13, 16, 20, 28].

Besides reduced physiologic reserve, many confounding factors like age, sex, timing of surgery as well as surgical expertise have been shown to influence postoperative outcome [1, 7, 29–33]. Current data suggests that the extent of gallbladder inflammation may equally affect postoperative outcomes as is the case with gangrenous and empyematous cholecystitis [34–37]. However, it is not known whether or not the high rates of complications associated with surgery for acute cholecystitis in critically ill patients is due to reduced physiologic reserve per se or to the extent of gallbladder inflammation. In this study, complicated cholecystitis (gangrenous, necrotizing and empyematous) was present in over 37 % of the study population compared to just 18 % of the control group.

Timing of surgical intervention has been a matter of debate for a long time. However, current evidence suggests a better outcome in patients managed within 72 h of symptom onset [1, 4, 38]. Although no statistically significant difference was seen amongst both groups with respect to the median time interval from diagnosis to surgery, surgery was performed later on patients with ASA scores > 2 as demonstrated by the interquartile range of 60 h in the study group. Indeed, delay cholecystectomy was performed in significantly more patients from the study group compared to the control group. This finding was not surprising since some of these patients required a correction of comorbid conditions prior to surgery.

There was no difference amongst both groups with regard to the rate of conversion. The rates of complications and mortality were significantly higher in the study group. Equally, the need for postoperative ICU management was significantly higher in the study groups. Although these findings are in accordance with current literature [39, 40], only 40 % of the study groups required ICU management in this series. This however is of little clinical implication, since the indication for ICU management varies amongst institutions.

With the exception of age, both groups were comparable in terms of baseline characteristics. This was also true for perioperative features in both groups. Therefore, the results recorded in this study cannot be blamed on these factors. Equally, surgical expertise was not an issue since both groups were managed by experienced surgeons. The results seen in this study therefore must be blamed either on reduced physiologic reserve per se or on the extent of gallbladder inflammation. However, since postoperative complications were recorded in a significant number of cases in patients with extensive gallbladder inflammation, the extent of inflammation rather than reduced physiologic reserve should be blamed for these complications.

The extent of gallbladder inflammation seems to depend on the duration of symptoms. Unequivocal evidence supporting the superiority of early cholecystectomy has become available over the last years [4, 5, 7, 8, 28, 41]. Delaying surgical intervention might be associated with worsening of inflammation. This seems to be the case with the study groups. The optimal time frame for surgery was most probably missed because of the need of specialist consultations and correction of comorbidities prior to surgery. Within this period, an initially uncomplicated cholecystitis probably got worse, resulting to a complicated form (gangrenous, empyematous or necrotizing cholecystitis).

In our department, same admission laparoscopic cholecystectomy represents the standard of care for patients with acute cholecystitis. Medical treatment is performed in a very small number of extremely morbid patients. Interventional methods like percutaneous cholecystostomy is not performed in our department. Therefore, it remains unclear, if and how the outcomes seen in this study would have been altered by medical and interventional treatment options.

Taken together, our results suggest that patients with reduced physiologic reserve presenting with acute cholecystitis are at increased risk for extensive gallbladder inflammation. The time spent on specialist consultation and for the correction of comorbidities prior to surgery forces such patients out of the optimal time frame for surgery. Delaying surgery in order to correct comorbidities may exacerbate the extent of inflammation, thereby leading to an increased risk of postoperative morbidity and the need of ICU management.

Our results argue for a timely management of critically ill patients with AC. First, surgery is best performed within 72 h of symptom onset. Second, medical treatment should be offered to patients who due to comorbidities cannot be managed within the safe time frame for surgery. However, the heterogeneity in the presentation and disease course in patients presenting with acute cholecystitis makes the clinical decision-making extremely difficult. This is especially true for patients who fail to recover on medical treatment and therefore have to undergo surgical management at the most unsuitable time. Therefore, the clinical decision-making must be individualized.

Although all patients included in this study were consecutive recruited and there was no risk of selection bias, the study design represents a major limitation of this study. Furthermore, it remains speculative, whether or not the outcomes recorded in this series might have been altered by medical or intervention treatment options. Thus, the trend reported in this series warrants further investigation.

## Conclusion

Critically ill patients presenting with acute cholecystitis are at increased risk for extensive gallbladder inflammation. The increased risk of morbidity and mortality seen in such patients might in part to be secondary to severe acute cholecystitis.

## Consent

Written informed consent was obtained from each patient for the publication of this study and any accompanying images.

## Abbreviation

AC: acute cholecystitis
ASA: American Society of Anesthesiologists
BMI: body mass index
CRP: C-reactive protein
ICU: intensive care unit
LC: laparoscopic cholecystectomy
LOS: length of stay
SPSS: statistical package for social sciences
WBC: white blood count
